# Estimating the escalating future need for palliative care among people living with dementia

**DOI:** 10.1177/02692163241269773

**Published:** 2024-09-12

**Authors:** Emel Yorganci, Anna E Bone, Catherine J Evans, Elizabeth L Sampson, Robert Stewart, Katherine E Sleeman

**Affiliations:** 1 Cicely Saunders Institute of Palliative Care, Policy & Rehabilitation, King’s College London, Florence Nightingale Faculty of Nursing, Midwifery & Palliative Care, London, UK; 2 Sussex Community NHS Foundation Trust, Brighton General Hospital, Brighton, UK; 3 Division of Psychiatry, University College London, London, UK; 4 Liaison Psychiatry, Royal London Hospital, East London NHS Foundation Trust, London, UK; 5 Centre for Psychiatry and Mental Health, Queen Mary’s University of London, London, UK; 6 Department of Psychological Medicine, Institute of Psychiatry, Psychology and Neuroscience, King’s College London, London, UK; 7 South London and Maudsley NHS Foundation Trust, London, UK

## Background

Globally, the number of people living and dying with dementia is escalating.^1,2^ People with dementia can have severe symptoms and complex care needs, throughout their illness trajectory, which can benefit from palliative care input.^3^ The fastest increase in serious health-related suffering by 2060 is expected to occur among people with dementia.^4^ Therefore, health and care systems across the world must be prepared to provide high-quality care to the increasing number of people affected by dementia. For this, up-to-date country-level estimates of the number of people with dementia who will have palliative care needs (decedents and non-decedents) are needed.^5,6^

Previous projections of the number of people with dementia who will have palliative care needs in England and Wales were based only on the number of people who died with dementia, and did not take into account people living with dementia.^7^ Thus, it is likely that the prevalence of palliative care needs among people with dementia in England and Wales has been considerably underestimated.

In England and Wales, recent estimates from Chen et al. show that by 2040 there will be 70% more people living with dementia than previously forecast.^8^ We aimed to estimate palliative care needs among people living with dementia in England and Wales to 2040, using recent projections on dementia prevalence.

## Methods

The Lancet Commission on Palliative Care and Pain Relief^6^ estimated that 40% of people living with dementia (at any stage) would benefit from palliative care. Chen et al. used data from the English Longitudinal Study of Ageing (ELSA),^9^ a longitudinal panel study of a representative sample of people aged 50 years or more living in England to estimate dementia prevalence to 2040. They used a multistate model accounting for non-linear trends in dementia incidence and bias, which also incorporated trends of mortality and disease incidence that reflect the composite trend of dementia risk factors to estimate dementia prevalence to 2040.^9^

We, therefore, applied the 40% multiplier to the dementia prevalence estimates obtained from Chen et al.’s projections (*dementia prevalence × 0.4* = *number of people with dementia who have palliative care needs*). This enables us to project the number of people living with dementia in England and Wales who will have palliative care needs by 2040. Chen et al. provided three separate models to project dementia prevalence. These assumed:

(i) Dementia incidence increases by 2.8% annually
(ii) Dementia incidence decreases by 2.7% annually
(iii) Dementia incidence remains constant over time

## Results

In all three models, the number of people living with dementia who will have palliative care needs is projected to substantially increase by 2040.

**Figure 1.**
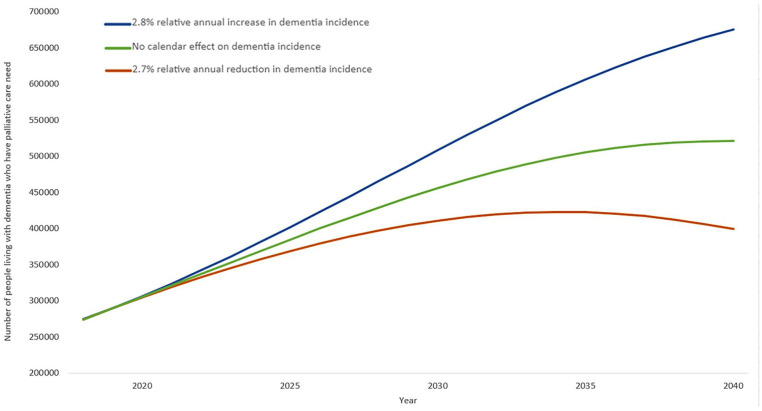
The projected number of people living with dementia in England and Wales who have palliative care needs, which was calculated using the primary scenario (Figure 4) from Chen et al.^8^

(i) Based on the most conservative model of dementia prevalence estimations, the number of people in England and Wales with dementia who have palliative care needs will increase from 274,000 in 2018 to 399,000 by 2040 (orange line in Figure 1).
(ii) Based on the model assuming that dementia incidence remains constant after 2018, the number of people with dementia in England and Wales who have palliative care needs is projected to increase to 521,000 by 2040 (green line in Figure 1).
(iii) Based on the model assuming an annual increase in dementia incidence, the number of people with dementia in England and Wales who have palliative care needs is projected to increase to 676,000 by 2040 (blue line in Figure 1).

## Discussion

Our analysis shows that even if dementia incidence declines between 2018 and 2040, the number of people living with dementia in England and Wales who have palliative care needs will increase substantially by 2040, reaching levels far greater than previous estimates based on mortality data.

Most palliative care for people with dementia is provided by generalist health and care professionals, including General Practitioners (GPs), community nurses and care home staff. Palliative care input can reduce symptom distress, as well as potentially burdensome healthcare use such as emergency department attendance and unplanned hospital admissions among people with dementia.^10–12^ Our data indicate that capacity for generalist and specialist palliative care for people with dementia should increase substantially in order to meet needs.

Studies that have projected palliative care needs typically rely on mortality data. For conditions such as dementia that are characterised by a trajectory of progressive deterioration over several years, projections relying on mortality data are likely to substantially underestimate needs. Application of similar methodologies in other countries would provide a better understanding of future palliative care needs. Projection methods for estimating palliative care needs among people with dementia which take into account fluctuating trajectories are needed.
